# Complement Fixing Polysaccharides from *Terminalia macroptera* Root Bark, Stem Bark and Leaves 

**DOI:** 10.3390/molecules19067440

**Published:** 2014-06-06

**Authors:** Yuan-Feng Zou, Bing-Zhao Zhang, Hilde Barsett, Kari Tvete Inngjerdingen, Drissa Diallo, Terje Einar Michaelsen, Berit Smestad Paulsen

**Affiliations:** 1 Department of Pharmaceutical Chemistry, School of Pharmacy, University of Oslo, P. O. Box 1068 Blindern, 0316 Oslo, Norway; 2 GIAT-HKU joint Center for Synthetic Biology Engineering Research (CSynBER), Guangzhou Institute of Advanced Technology, Chinese Academy of Sciences, 511458 Nansha, Guangzhou, China; 3 Department of Traditional Medicine, BP 1746, Bamako, Mali

**Keywords:** *Terminalia macroptera*, accelerated solvent extraction, polysaccharides, complement fixation activity

## Abstract

The root bark, stem bark and leaves of *Terminalia macroptera* were sequentially extracted with ethanol, 50% ethanol-water, and 50 °C and 100 °C water using an accelerated solvent extractor. Ten bioactive purified polysaccharide fractions were obtained from those crude extracts after anion exchange chromatography and gel filtration. The polysaccharides and their native extracts were characterized with respect to molecular weight, chemical compositions and effects in the complement assay. The chemical compositions showed that the polysaccharides are of pectic nature. The results indicated that there was no great difference of the complement fixation activities in the crude extracts from the different plant parts when extracting with the accelerated solvent extraction system. The purified polysaccharide fractions 100WTSBH-I-I and 100WTRBH-I-I isolated from the 100 °C water extracts of stem and root bark respectively, showed the highest complement fixation activities. These two fractions have rhamnogalacturonan type I backbone, but only 100WTSBH-I-I contains side chains of both arabinogalactan type I and II. Based on the yield and activities of the fractions studied those from the root bark gave highest results, followed by those from leaves and stem bark. But in total, all plant materials are good sources for fractions containing bioactive polysaccharides.

## 1. Introduction

*Terminalia macroptera* Guill. & Perr. (Combretaceae) is a tree, up to 20 m high, which occurs widely in West Africa. In Mali *T. macroptera* is used against a variety of ailments, and more than 30 different indications have been mentioned by the traditional healers in ethnopharmacological studies. The stem bark and leaves are most commonly used against sores and wounds, pain, cough, tuberculosis and hepatitis [1]. The roots are used against hepatitis, gonorrhea and various infectious diseases, including *H. pylori*-associated diseases [1,2,3,4,5]. Flavonoids [6,7,8], triterpenoids [9,10], ellagitannins [11] and related phenolics [3,9,12], have been identified from different parts of *T. macroptera*. Ellagitannins are known antimicrobial compounds which may be related to the use of the leaves against wounds and infections [13]. Water decoctions of *T. macroptera*, administered orally, are the most common preparations used by the traditional healers in Mali [1]. Plant polysaccharides isolated from crude water extracts have shown effects related to the immune system by different *in vitro* and *in vivo* test systems [14]. The chemical characteristics and biological activities of polysaccharides, especially those from plants used in the treatment of wounds, ulcer and cancer have been reported [15,16,17,18,19]. Thus, it was of interest to investigate the bioactive polysaccharides from water extracts of *T. macroptera*.

Traditionally in laboratory studies, low molecular weight and lipophilic compounds are extracted from plant material by the Soxhlet extraction method. Accelerated solvent extraction (ASE) for these type of compounds was first described in 1995 [20,21], and the method has grown steadily in use since that time [22]. Under elevated temperature and pressure, an extraction solvent can be used above its boiling point but still remain in the liquid state, and thus increasing the kinetics of the extraction process. In this case, solvent consumption and extraction times are significantly decreased [23]. ASE has been applied for extracting components from environment samples [24,25,26,27], biological materials [28], plant materials [29,30,31,32,33], dietary compounds [34], feeds [35,36], and food [37,38]. However, reports on the use of ASE for polysaccharide extraction mainly from woods [39,40,41] have only recently been reported. Thus, it was of interest to investigate the isolation of bioactive polysaccharides from medicinal plant by ASE.

Root bark, stem bark and leaves are used in traditional medicine in Mali against several ailments; among those are illnesses where the immune system is involved quite frequent. Due to the use of all plant parts, it was relevant to investigate if polysaccharides from the three different plant parts had similar type of bioactivity and structural features. If this was the case, the recommendations should be to rather use the leaves than the other plant parts as this will lead to a more sustainable use of the tree. If e.g., roots are overused, eradication of trees may be the result. Therefore, in this study, ASE was employed to extract polysaccharides from root bark, stem bark and leaves from *T. macroptera*. The aims of this study are comparison of the properties of the polysaccharides from the different plant parts, as well as relationships between the chemical characteristics and complement fixation activities. Crude polysaccharide extracts were obtained and further purified, the chemical characteristics and complement fixation activities of polysaccharide fractions were evaluated, and the results from the three different plant parts were compared.

## 2. Results and Discussion

### 2.1. Crude Extracts

#### 2.1.1. Yields

The yields of the crude water extracts from the root bark (50WTRBH and 100WTRBH) obtained from ASE described, were 0.6% and 2.5% respectively. The yields of the crude water extracts from stem bark (50WTSBH and 100WTSBH) were 0.5% and 1.3%. The yields of the crude water extracts from leaves (50WTLH and 100WTLH) were 0.3% and 0.6%. All of the yields given in brackets in Table 1 are related to the dried, powdered materials. Extraction with ASE showed that the root bark gave higher yields of crude extracts than what was obtained from the stem bark and leaves.

**Table 1:** Monosaccharide compositions of crude water extracts from root bark, stem bark and leaves of *T. macroptera*.

	50WTRBH	100WTRBH	50WTSBH	100WTSBH	50WTLH	100WTLH
Ara ^a^	5.9	0.4	14.0	0.6	15.3	10.2
Rha ^a^	11.8	0.6	9.5	1.3	7.7	2.4
Xyl ^a^	2.1	0.3	4.7	0.7	3.3	2.9
Man ^a^	1.0	0.2	0.6	Trace	2.0	7.8
Gal ^a^	20.2	1.1	23.7	1.3	18.5	8.4
Glc ^a^	36.5	97.4	23.3	92.1	14.8	49.8
GlcA ^a^	1.0	n.d.	4.3	Trace	2.6	Trace
GalA ^a^	21.4	Trace	19.9	4.0	35.8	18.4
Yield (% w/w) ^b^	0.6	2.5	0.5	1.3	0.3	0.6
Presence of starch	+	+	+	+	+	+

^a^ mol% related to total content of the monosaccharides Ara, Rha, Xyl, Man, Gal, Glc, GlcA and GalA; ^b^ yield related to the dried, powdered materials; n.d. not detected.

#### 2.1.2. Chemical Compositions

After methanolysis, the monosaccharide compositions of the crude water extracts were analyzed by GC as the TMS derivatives of the methyl-glycosides. As can be seen from Table 1, the monosaccharide compositions of extracts from 50 °C water have similarities, but also differences. The compositions of the polymers in 50WTRBH and 50WTSBH and 50WTLH are quite similar, containing the neutral monosaccharides arabinose (Ara), rhamnose (Rha), galactose (Gal) and glucose (Glc), albeit in different amounts. In addition to the neutral monosaccharides, the 50WTRBH, 50WTSBH and 50WTLH also contain galacturonic acid (GalA). The crude extracts 100WTRBH and 100WTSBH contain more than 90 mol% of Glc. The glucose detected in the crude extracts most probably comes from starch as the iodine test gave a strong positive reaction (Table 1). 

#### 2.1.3. Complement Fixation Activity

The complement system is an important part of the immune defense, such as primary defense against bacterial invasions and viral infections. Complement fixating activity of polysaccharides from plants has previously been shown as an indicator for effects on the immune system [42,43].

**Figure 1:**
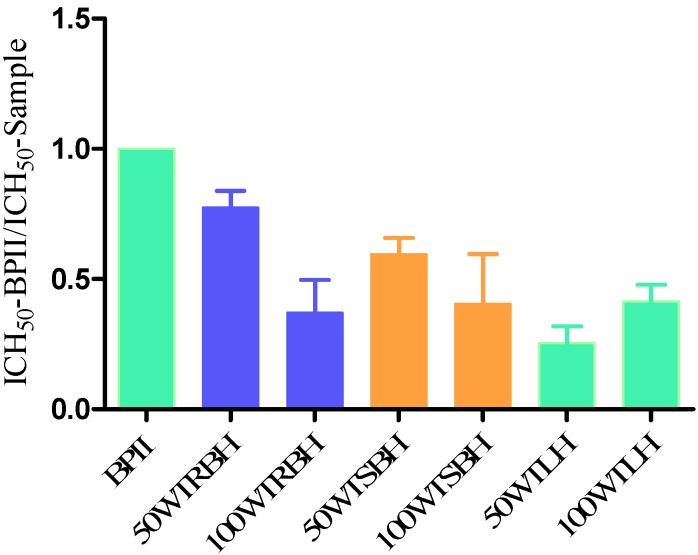
Complement-fixating activities of the crude water extracts obtained by ASE from root bark, stem bark and leaves of *T. macroptera* related to positive control (BP II from *Biophytum petersianum*). ICH_50_-BPII/ ICH_50_-Sample shows how active each individual test sample is compared to the positive control BPII.

As can be seen from Figure 1, the crude water extracts from ASE of root bark, stem bark and leaves showed potent human complement fixation activities *in vitro*. The activity of the crude water extract 50WRB was slightly higher than other crude extracts, but all have slightly lower activities than the very active, positive control BPII. The crude water extracts from 100 °C of root bark (100WTRBH), stem bark (100WTSBH) and leaves (100WTLH) showed similar complement fixation activities.

### 2.2. Studies on Purified Polysaccharide Fractions

The crude extracts were further purified by ion exchange chromatography and the isolated sub-fractions with activity in the complement fixation assay, were subjected to gel filtration. The purified fractions thus obtained where the objects for further studies as given below. 

#### 2.2.1. Yields

Three active purified polysaccharide fractions, 50WTRBH-I-I, 50WRBH-II-I and 100WRBH-I-I, were obtained from root bark crude extracts. The stem bark crude water extracts gave four active fractions; 50WTSBH-I-I and 50WTSBH-II-I were isolated from 50WTSBH and two 100WTSBH-I-I and 100WTSBH-III-I from 100WTSBH. Using the same purification procedure for the leaf polysaccharides the following fractions were obtained: two from 50WTLH (50WTLH-I-I and 50WTLH-II-I) and one from 100WTLH (100WTLH-I-I). The total yields of purified polysaccharide fractions from stem bark were higher than those from root bark and leaves. All of the yields given in Table 2 are based on the dried, powdered materials.

#### 2.2.2. Chemical Compositions

The characterization of the purified polysaccharide fractions are given in Table 2. All the purified fractions contain the monosaccharides that are typical constituents in pectic polysaccharides (Table 2). The presences of AG-II in the root bark polysaccharides 50WTRBH-I-I and 50WTRBH-II-I and all the purified active polysaccharide fractions from the stem bark and leaves were identified by the Yariv-test. Purification of the polysaccharide fractions gave a huge reduction in the content of Glc. Still some purified fractions (100WTRBH-I-I, 100WTSBH-I-I, 100WTSBH-III-I and 100WTLH-I-I) from 100 °C water extracts have rather high amounts of Glc which most probably comes from starch, as positive reactions were observed in the iodine test. The monosaccharide compositions of the purified polysaccharide fraction 100WTRBH-I-I were quite different from other fractions, such as absence of Ara, Xyl and GlcA, and higher amount of Glc.

The Bio-Rad protein assay showed negligible amounts (<1%) of protein present in the fractions. Phenolic compounds were found in fraction 100WTSBH-III-I (3.0%), determined by the Folin-Ciocalteu assay, while only minor amounts were present in fractions 50WTSBH-I-I, 50WTLH-I-I and 100WTLH-I-I. Totally, the phenolic contents of the purified fractions from stem bark were slightly higher than those from leaves, while none was detected in the fractions from root bark. 

#### 2.2.3. Molecular Weight Distribution

Size exclusion chromatography, using dextran standards, was employed to determine the average *M_W_* of the purified fractions. The highest molecular weight was found in the fraction 100WRB-I-I (491.8 kDa), while the lowest molecular weight (19.8 kDa) was found in the fractions 50WTLH-I-I and 50WTSBH-I-I. Generally, the molecular weights of most of the purified fractions from root bark and leaves are higher than those from stem bark.

#### 2.2.4. Complement Fixation Activity

**Figure 2:**
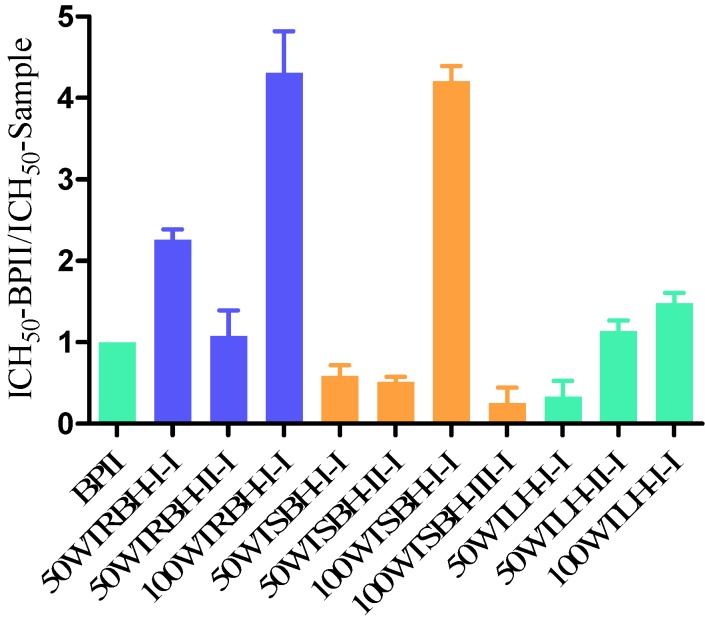
Complement-fixating activities of purified polysaccharide fractions obtained from root bark, stem bark and leaves of *T. macroptera* related to positive control BPII. ICH_50_-BPII/ ICH_50_-Sample shows how active each individual test sample is compared to the positive control BPII.

As can be seen from Figure 2, the purified polysaccharide fractions showed potent human complement fixation activities *in vitro*. After fractionation, all purified polysaccharide fractions from the root bark, showed higher activity compared to the positive control BPII, the fraction 100WTRBH-I-I being the most potent. The fractions from stem bark were potent, but only fraction 100WTSBH-I-I was more active on weight basis than BPII. Considering the purified fractions from leaves, 50WTLH-II-I and 100WTLH-I-I showed higher activity than BPII, and 100WTLH-I-I was the most potent one. 

Totally, among the purified fractions, 100WTRBH-I-I and 100WTSBH-I-I, were the most potent fractions, showing almost the same activity. The other fractions from root bark were more active than the fractions from stem bark. The fractions 50WTRBH-I-I and 100WTRBH-I-I from root bark showed both higher activities than the purified fractions from leaves. 

**Table 2:** Characterizations of polysaccharide fractions isolated from root bark, stem bark and leaves of *T. macroptera* after ion exchange chromatography and gel filtration.

	50WTRBH-I-I	50WTRBH-II-I	100WTRBH-I-I	50WTSBH-I-I	50WTSBH-II-I	100WTSBH-I-I	100WTSBH-III-I	50WTLH-I-I	50WTLH-II-I	100WTLH-I-I
Ara ^a^	19.6	7.1	n.d.	15.4	10.8	16.4	9.7	15.2	11.7	25.4
Rha ^a^	3.5	29.1	23.6	4.3	18.4	4.3	14.4	2.3	12.5	3.1
Xyl ^a^	6.4	2.3	n.d.	21.4	4.3	1.6	4.2	34.8	1.5	23.9
Man ^a^	1.0	2.2	1.0	2.6	0.4	0.8	n.d.	2.7	0.3	4.1
Gal ^a^	48.3	15.3	3.7	34.7	16.2	19.3	7.5	21.2	10.7	23.6
Glc ^a^	2.1	1.6	25.8	3.6	2.7	19.0	9.5	7.1	2.2	7.2
GlcA ^a^	2.1	3.1	n.d.	2.8	3.4	1.7	4.9	0.9	5.9	0.3
GalA ^a^	17.0	39.2	45.9	15.1	43.6	36.9	49.9	15.8	55.2	12.4
Yield (% w/w) ^b^	0.001	0.01	0.01	0.003	0.014	0.003	0.033	0.003	0.016	0.01
Mw (kDa)	136.1	115.9	491.8	19.8	23.2	98.7	44.2	19.8	220.3	136.1
The Yariv test ^c^	++	++	-	+	++	++	++	+	++	+
Presence of starch	-	-	+	-	-	+	+	+	-	+
Protein (% w/w)	0.1	n.d.	n.d.	0.9	n.d.	n.d.	0.7	0.3	0.2	n.d.
Phenols (% w/w) ^d^	n.d.	n.d.	n.d.	0.4	n.d.	n.d.	3.0	2.1	n.d.	0.6

^a^ mol% related to total content of the monosaccharides Ara, Rha, Xyl, Man, Gal, Glc, GlcA and GalA; ^b^ yield related to the dried, powdered material; ^c^ The presence of arabinogalactans type II (AG-II) was identified by precipitation with the β-glycosyl Yariv reagent; ^d^ The total phenolic content is expressed as ferulic acid equivalents; n.d. not detected.

#### 2.2.5. Linkage Analysis of the Polysaccharide Fractions

The most active fractions from each plant part, 100WTRBH-I-I from root bark, 100WTSBH-I-I from stem bark and 100WTLH-I-I from leaves, were selected for linkage analysis. In order to determine the nature of the glycosidic linkages of the different monosaccharides in the purified fractions, permethylation of the reduced polymers was performed, partially O-methylated alditol acetates (PMAAs) were prepared and subjected to GC-MS. The results are given in Table 3.

The main structural feature of 100WTSBH-I-I and 100WTLH-I-I are similar, having 1,4-linked galacturonan, with a few branch points in position 3 of GalA. The Rha units are 1,2-linked, with branch points on position 4. The low ratio of Rha to GalA indicate that the backbone of the polysaccharide fractions consist of shorter RG-I structures, and longer homogalacturonan regions. The Gal and Ara present have the normal type of linkages that are found in the AG II side chain [44]. The presence of 1,3-linked and 1,3,6-linked Gal indicate the presence of AG-II structures in these two fractions, as they gave positive reactions in the Yariv test. The presence of 1,4-linked Gal may indicate the presence of AG-I in these two fractions [14]. The Xyl is present as 1,4-linked unit in fraction 100WTL-I-I, and lower amount was present in 100WTSBH-I-I. These features have certain similarities with pectins that are composed of areas with hairy or ramified and smoother regions [45]. GlcA appearing in these two fractions are mainly terminally linked units. Terminal GlcA might be directly linked to position 3 of 1,4-linked GalA in the RG-I backbone, or may also be a part of the AG-II side chains [46,47]. 

The structural feature of 100WTRBH-I-I was quite different from other two fractions. The main structural feature of 100WTRBH-I-I is the presence of RG-I, but without AG-I/II since Ara is absent and only a low amount of Gal is found. The higher ratio of 1,2-linked Rha to 1,4-linked GalA indicate that the backbone of the 100WTRBH-I-I consists of longer RG-I structures, and shorter homogalacturonan regions. High amount of Glc, mainly as 1,4-linked and small amount of 1,4,6-linked may indicate that they come from starch as positive reactions were observed in the iodine test. This could be due to a physical binding between RG-I backbone with a few Gal units and starch bound so tightly that they could not be separated by the method used.

**Table 3:** The linkages (mol%) of the monosaccharides present in the most active purified fractions from root bark, stem bark and leaves of T. macroptera determined by GC–MS after methylation.

		100WTRBH-I-I	100WTSBH-I-I	100WTLH-I-I
Ara	T*f*	n.d.	10.2	14.6
	1,2*f*	n.d.	0.1	1.2
	1,3*f*	n.d.	0.2	0.7
	1,5*f*	n.d.	4.3	6.7
	1,3,5*f*	n.d.	1.6	2.1
Rha	T*p*	n.d.	1.5	1.3
	1,3*p*	1.3	0.1	0.1
	1,2*p*	21.3	0.5	n.d.
	1,2,4*p*	1.0	2.2	1.7
Xyl	T*p*	n.d.	n.d.	Trace
	1,4*p*	n.d.	1.5	23.8
Gal	T*p*	1.2	3.4	2.1
	1,4*p*	n.d.	0.5	3.3
	1,3*p*	2.1	1.2	4.8
	1,6*p*	n.d.	9.9	2.1
	1,3,6*p*	0.4	3.1	10.7
	1,3,4,6*p*	n.d.	0.6	0.3
Glc	1,3*p*	n.d.	0.7	2.3
	1,4*p*	21.8	13.9	3.5
	1,6*p*	4.0	3.8	0.7
	1,4,6*p*	Trace	0.4	0.5
GlcA	T*p*	n.d.	1.0	0.3
	1,4*p*	n.d.	0.7	n.d.
GalA	T*p*	1.3	n.d.	n.d.
	1,4*p*	44.6	34.6	12.3
	1,3,4*p*	n.d.	2.3	0.1

### 2.3. Discussion

*T. macroptera* root bark, stem bark and leaves are used against a variety of ailments, such as against sores and wounds, pain, cough, tuberculosis and hepatitis [1]. Medicinal plants used for wound healing often appear to be rich in polysaccharides, which may be responsible of their wound healing properties [48]. The ASE is a highly efficient extraction method which significantly reduce the extraction time and amount of solvents used. It was therefore of interest to study the structure and biological activity of polysaccharides from root bark, stem bark and leaves of *T. macroptera* obtained by ASE. 

The chemical and biological characteristics of crude water extracts from different plant parts had some similarities, but also differences. All the crude water extracts showed similar potent complement fixation activities. All the crude extracts were fractionated by ion exchange chromatography and gel filtration as described, and led to the isolation of ten active sub-fractions with different molecular weights. The chemical compositions of these sub-fractions were quite different (Table 2), but all contained monosaccharides typical for pectic type polysaccharides. The purification procedure induces a huge amount of Glc in all fractions, which lead the increase of complement fixation activities after removal of the glucose polymers. But still some Glc polymer were present in some of the fractions, this could be due to a RG-I backbone this could be due to a RG-I backbone with a few Gal units attached in addition to starch, that physically bound tightly to the polymer and could not be removed by the method used.

It has been reported that acidic polysaccharides with higher molecular weights appear to be more active in the complement assay than those with lower molecular weights [49,50,51]. Among the ten sub-fractions in our present study, fraction 100WTRBH-I-I with the highest molecular weight exhibited the highest activity, but the other fractions with different molecular weight did not follow this trend.

In addition to molecular weight differences, the type of monosaccharide linkages might be another reason for the influence on the complement fixation activity. Pectins are generally known to be composed of linear homogalacturonan (HG) regions and branched rhamnogalacturonan (RG) I and II regions [52]. The side chains of RG-I consist usually of arabinogalactan (AG) type I and/or II, as well as arabinan and galactan. The branched regions of the pectins are thought to be related to their immunomodulating activities. Polysaccharides rich in AG-II have shown effects in a number of biological assays [14,18,49,53]. The results of the Yariv-test showed that all purified active fractions contain AG-II structures, except 100WTRBH-I-I from the root bark. The fraction 100WTLH-I-I is highly ramified compared to other two fractions. As the RG-I backbone generally consists of alternating units of Rha and GalA, the low Rha to GalA ratio indicates that the backbone of the polysaccharide fractions consist of shorter RG-I structures, and longer homogalacturonan regions. RG-I regi ith a few Gal units attached in addition to starch, that physically bound tightly to the polymer and could not be removed by the method used.

It has been reported that acidic polysaccharides with higher molecular weights appear to be more active in the complement assay than those with lower molecular weights [49,50,51]. Among the ten sub-fractions in our present study, fraction 100WTRBH-I-I with the highest molecular weight exhibited the highest activity, but the other fractions with different molecular weight did not follow this trend.

In addition to molecular weight differences, the type of monosaccharide linkages might be another reason for the influence on the complement fixation activity. Pectins are generally ons were present in all fractions, but with different lengths. Sub-fraction 100WTRBH-I-I may contain longer RG-I region than other sub-fractions as deducted from the linkages shown in Table 3. Sub-fractions 100WTSBH-I-I and 100WTLH-I-I may contain AG-I due to presence of 1,4-linked Gal units. The structural feature of positive control BPII is 1,4-linked galacturonan with branching on position 3, interrupted by RG-I region, side chains (AG-II) branched on position 4 of Rha [54]. The main structural features of 100WTSBH-I-I and 100WTLH-I-I are similar, having 1,4-linked galacturonan with branching on position 3, interrupted by RG-I region, side chains (AG-I and AG-II) branched on position 4 of Rha. The structural feature of fraction 100WTRBH-I-I is 1,4-linked galacturonan, interrupted by a long RG-I region, side chains (galactan) branched on position 4 of Rha. These similarities between BPII and our polysaccharide fractions may explain the possibility of our samples exhibited potent complement fixation activities. In addition, our samples, 100WTRBH-I-I (491.8 kDa), 100WTSBH-I-I (98.7 kDa) and 100WTLH-I-I (136.1 kDa), has higher *Mw* than BPII (31kDa), which may explain why they showed stronger complement fixation activities than BPII. 

Various immunomodulating polysaccharides isolated from plants (*Opilia celtidifolia*, *Vernonia kotschyana, Brassica oleracea*) also contain protein and phenolic compounds [51,55,56]. Many phenolic compounds were showed with potent complement activity, as reviewed by Pieters [57]. The presence of phenolic substances in the purified fractions might be due to ferulic acid being linked as ester to Ara and Gal in pectins [58]. The sub-fractions 100WTSBH-III-I and 50WTLH-I-I contain higher amounts of protein and phenolic compounds than other sub-fractions, which may explain part of the observed complement fixation activity in the lower molecular weight sub-fractions. 

## 3. Experimental

### 3.1. Plant Material

The root bark, stem bark and leaves of *T. macroptera* were collected in Mali, December 2011, and identified by the Department of Traditional Medicine (DMT), Mali. A voucher specimen is deposited at the herbarium of DMT (Voucher No. 2468/DMT). The plant material was washed, cut into small pieces, dried and pulverized to a fine powder by a mechanical grinder. 

### 3.2. Extraction of Polysaccharides

**Figure 3:**
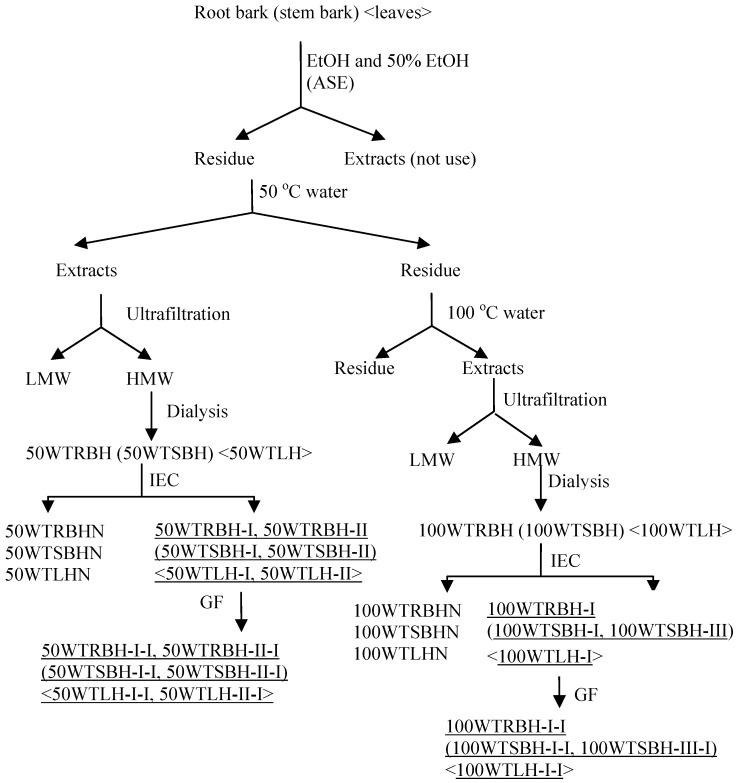
Extraction and fractionation scheme of polysaccharides extracted by accelerated solvent extraction (ASE) from root bark, stem bark and leaves of *T. macroptera* (IEC, ion exchange chromatography; GF, gel filtration; underlined acidic fractions showed high complement fixation activity and were fractionated for further studies).

ASE was performed on a Dionex ASE350 Accelerated Solvent Extractor (Dionex, Sunnyvale, CA, USA). Powdered root bark, stem bark or leaves (200 g of each) were weighed and mixed with diatomaceous earth (50 g), and then packed in eight 100 mL stainless steel cells. The extractions were performed at 1,500 psi, with 5 min heating, 5 min static time, and a 60 s purge for a total of three cycles. In order to remove low molecular weight compounds, the cells were pre-extracted twice with 96% ethanol (EtOH) at 70 °C. The cells were further extracted twice with 50% ethanol-water at 70 °C, followed by distilled water of 50 °C and 100 °C. After extraction the water extracts were subjected to ultrafiltration (cut off 5,000 Da), and the high molecular weight (HMW) fraction was concentrated and dialyzed at cut-off 3,500 Da, lyophilized and kept for further studies. The crude water extracts were named 50WRBH and 100WRBH for the root bark, and 50WSBH and 100WSBH for the stem bark, and 50WTLH and 100WTLH for leaves (Figure 3). These fractions were subjected to determination of monosaccharide composition, starch content investigation and complement fixation assay.

### 3.3. Fractionation and Characterization of Polysaccharides

The crude extracts were further fractionated by anion exchange and gel filtration. All purified fractions were subjected to determination of their chemical and biological characteristics. 

#### 3.3.1. Ion Exchange Chromatography and Gel Filtration

The crude extracts from ASE were filtered through 0.45 μm filters and applied to an anion exchange column packed with ANX Sepharose™ 4 Fast Flow (high sub) (GE Healthcare, Uppsala, Sweden). The neutral fractions were eluted with distilled water at (2 mL/min), while the acidic fractions were eluted with a linear NaCl gradient in water (0–1.5 M) at 2 mL/min. The carbohydrate elution profiles were monitored using the phenol-sulfuric acid method [59]. The related fractions were pooled, dialyzed at cut-off 3500 Da against distilled water for removal of NaCl, and lyophilized (Figure 3) 

The acidic fractions marked in Figure 3 were dissolved in elution buffer (10 mM NaCl), filtered through a Millipore filter (0.45 μm), and subjected to gel filtration after application on a Hiload™ 26/60 Superdex™ 200 prep grade column (GE Healthcare) combined with the Äkta system (FPLC, Pharmacia Äkta, Amersham Pharmacia Biotech, Uppsala, Sweden), and eluted with 10 mM NaCl at 1.0 mL/min. Fractions were pooled based on their elution profiles, as determined by the phenol-sulfuric acid method, dialyzed and lyophilized.

#### 3.3.2. Determination of Monosaccharide Composition

The monosaccharide compositions of the crude extracts and purified fractions were determined by gas chromatography of the trimethylsilylated (TMS) derivatives of the methyl-glycosides obtained after methanolysis with 3 M hydrochloric acid in anhydrous methanol for 24 h at 80 °C [60,61,62]. Mannitol was used as an internal standard. The TMS derivatives were analyzed by capillary gas chromatography on a Focus GC (Thermo Scientific, Milan, Italy).

#### 3.3.3. Test for the Presence of Starch

The presence of starch in the fractions was tested by adding two drops of an aqueous iodine-potassium-iodide solution to the samples [63]. A positive reaction gives a dark bluish color. Starch was used as a positive control.

#### 3.3.4. Molecular Weight Determination

The homogeneity and molecular weight of the purified polysaccharide fractions were determined by size exclusion chromatography on a Hiload™ 16/60 Superdex™ 200 prep grade column (GE Healthcare) combined with the Äkta system (FPLC, Pharmacia Äkta). Dextran polymers (Pharmacia) B512 (5.6 kDa), T8360 (19 kDa), T250 (233 kDa) and T500 (475 kDa) were used as calibration standards. Approximately 5 mg of the samples were dissolved in 2 mL of 10 mM NaCl buffer and filtered through a Millipore filter (0.45 μm) and applied to the column. The samples were eluted with 10 mM NaCl at 0.5 mL/min, collecting 2 mL fractions. The eluent was detected with a Shimadzu RI detector. The retention volume was converted to molecular weight by using the standards.

#### 3.3.5. Precipitation with the Yariv β-Glucosyl Reagent

Precipitation with the Yariv β-glucosyl reagent was performed on the samples as described by van Holst and Clarke [64]. The Yariv β-glucosyl reagent forms a colored precipitate with compounds containing AG-II structures. A solution of Arabic gum in water (1 mg/mL) was used as a positive control.

#### 3.3.6. Determination of Phenolic Content

The total amount of phenolic compounds in the purified polysaccharide fractions were quantitatively determined using the Folin-Ciocalteu assay [65]. 200 microliter sample (1 mg/mL) dissolved in water (three replicates) was added the same amount of Folin-Ciocalteu’s phenol reagent (1:1 in water, Merck/Kebo), mixed and left for 3 min at room temperature. 200 microliter of 1 M Na_2_CO_3_ was added; the tubes were mixed and allowed to stand for 1 h. The absorbance was measured at 750 nm. A standard curve was plotted using ferulic acid. The total phenolic content was determined as ferulic acid equivalents (FA/sample) × 100%.

#### 3.3.7. Determination of Protein Content

The protein content of the polysaccharide fractions was determined by the Bio-Rad protein assay, based on the method of Bradford (Bio-Rad, Hercules, CA, USA) [66]. The standard procedure for microliter plates was used with bovine serum albumin (BSA) as a protein standard in a concentration range from 15 to 500 μg/mL. The Bio-Rad protein assay is a dye-binding assay in which a differential color change of a dye occurs in response to various concentrations of protein. The absorbance maximum for an acidic solution of Coomassie^®^ Brilliant Blue G-250 dye shifts from 465 nm to 595 nm when binding to protein occurs.

#### 3.3.8. Complement Fixation Assay

The complement system is an important part of the innate immune system which also cooperates with the adaptive immune system in many ways [67]. Complement among other things play a direct part in the defence, such as primary defence against bacterial invasions and viral infections. Complement fixating activity of polysaccharides from plants has previously been used as an indicator for effect on the immune system [42,43]. Many resent publications support the notion that pectins convey at least some of the beneficial effect of medicinal plants and that this might be based on the interaction with the immune system [18]. 

The complement fixation test is based on inhibition of hemolysis of antibody sensitized sheep red blood cells (SRBC) by human sera as described by Michaelsen *et al.* (Method A) [42]. It is a quick, highly reproducible assay performed in microtiter plates with many samples analysed simultaneously and with positive control. BPII, a highly active pectic polysaccharide from the aerial parts of *Biophytum petersianum* Klotzsch (also known as *B. umbraculum*) [68], was used as a positive control. The indicator system in the assay is inhibition of haemolysis induced by human complement. Samples showing inhibition in the assay is thus having a direct effect on the human immune system. Inhibition of lysis induced by the test samples was calculated by the formula [(Acontrol-Atest)/Acontrol] × 100%. From these data, a dose-response curve was created to calculate the concentration of test sample giving 50% inhibition of lysis (ICH_50_). A low ICH_50_ value means a high complement fixation activity. The activity of all the polysaccharide fractions are given as the ICH_50_ value of the positive control BP-II divided on the ICH_50_ value of the sample. 

#### 3.3.9. Determination of Glycosidic Linkages

Glycosidic linkage elucidation was performed by methylation studies. The most active fractions from each plant part were selected for methylation. Prior to methylation, the free uronic acids were reduced with NaBD_4_ to their corresponding neutral sugars. After reduction of the polymers, methylation, hydrolysis, reduction and acetylation [69] were carried out. The derivatives were analyzed by GC-MS using a GCMS-QP2010 (Shimadzu, Kyoto, Japan) attached to a Restek Rxi-5MS (30 m; 0.25 mm i.d.; 0.25 µm film) column. The injector temperature was 280 °C, the ion source temperature 200 °C and the interface temperature 300 °C. The column temperature was 80 °C when injected, then increased with 10 °C/min to 140 °C, followed by 4 °C/min to 210 °C and then 20 °C/min to 300 °C. Helium was the carrier gas (pressure control: 80 kPa.) The compound at each peak was characterized by an interpretation of the retention times and the characteristic mass spectra. The estimation of the relative amounts of each linkage type was related to the total amount of each monosaccharide type as determined by methanolysis. Effective carbon-response factors were applied for quantification [70].

## 4. Conclusions

The root bark, stem bark and leaves of *T. macroptera* were traditionally used against wounds and various infection diseases. All these ailments involve the immune system. Ten purified polysaccharide fractions were isolated from six different crude water extracts from root bark, stem bark and leaves. These polysaccharide fractions all exhibited potent complement fixation activities. The complement system plays a direct part in the defense, such as primary defense against bacterial invasions and viral infections. Therefore, the traditional use of this tree to against wounds and various infection diseases may be, at least partly, connected to the complement system. Comparing the polysaccharide fractions from different plant parts, it is clear that they have some similarities when it comes to biological activity and structures, although some differences are present. In summary, the root bark, stem bark and leaves are all good sources for fractions containing bioactive polysaccharides. But due to sustainability of the tree *T. macroptera*, the authors would recommend that leaves should be used as a traditional remedy against illnesses where the immune system is involved instead of root bark or stem bark, but then higher dosage by weight has to be used. However, before plant part replacement should be recommended, it is important to also address the secondary metabolites as possible active components. Moreover, further studies should be focused on the active sites of the bioactive polysaccharides connected to the complement system, as well as in other biological assay.

## Abbreviations

AG-I: arabinogalactan type I
AG-II: arabinogalactan type II
Ara: arabinose
ASE: accelerated solvent extraction
DCM: dichloromethane
EtOH: ethanol
Gal: galactose
GalA: galacturonic acid
Glc: glucose
GlcA: glucuronic acid
GF: gel filtration
HMW: high molecular weight
IEC: ion exchange chromatography
LMW: low molecular weight
Man: mannose
MeOH: methanol
RG-I: rhamnogalacturonan type I
Rha: rhamnose
Xyl: xylose
